# COVID-19: Seroprevalence and Vaccine Responses in UK Dental Care Professionals

**DOI:** 10.1177/00220345211020270

**Published:** 2021-06-02

**Authors:** A.M. Shields, S.E. Faustini, C.A. Kristunas, A.M. Cook, C. Backhouse, L. Dunbar, D. Ebanks, B. Emmanuel, E. Crouch, A. Kröger, J. Hirschfeld, P. Sharma, R. Jaffery, S. Nowak, S. Gee, M.T. Drayson, A.G. Richter, T. Dietrich, I.L.C. Chapple

**Affiliations:** 1 Clinical Immunology Service, Institute for Immunology and Immunotherapy, University of Birmingham, Birmingham, UK; 2 Institute of Clinical Sciences, University of Birmingham, Birmingham, UK; 3 The Binding Site Group Ltd, Birmingham, UK; 4 Birmingham Local Dental Committee, Birmingham, UK; 5 Birmingham Community Healthcare NHS Foundation Trust, Birmingham, UK; 6 Department of Oral Surgery, The School of Dentistry, University of Birmingham, Birmingham, UK; 7 Periodontal Research Group, University of Birmingham, Birmingham, UK

**Keywords:** dentistry, SARS-CoV-2, vaccination, antibodies, seroepidemiological studies, occupational exposure

## Abstract

Dental care professionals (DCPs) are thought to be at enhanced risk of occupational exposure to severe acute respiratory syndrome coronavirus 2 (SARS-CoV-2). However, robust data to support this from large-scale seroepidemiological studies are lacking. We report a longitudinal seroprevalence analysis of antibodies to SARS-CoV-2 spike glycoprotein, with baseline sampling prior to large-scale practice reopening in July 2020 and follow-up postimplementation of new public health guidance on infection prevention control (IPC) and enhanced personal protective equipment (PPE). In total, 1,507 West Midlands DCPs were recruited into this study in June 2020. Baseline seroprevalence was determined using a combined IgGAM enzyme-linked immunosorbent assay and the cohort followed longitudinally for 6 mo until January/February 2021 through the second wave of the coronavirus disease 2019 pandemic in the United Kingdom and vaccination commencement. Baseline seroprevalence was 16.3%, compared to estimates in the regional population of 6% to 7%. Seropositivity was retained in over 70% of participants at 3- and 6-mo follow-up and conferred a 75% reduced risk of infection. Nonwhite ethnicity and living in areas of greater deprivation were associated with increased baseline seroprevalence. During follow-up, no polymerase chain reaction–proven infections occurred in individuals with a baseline anti–SARS-CoV-2 IgG level greater than 147.6 IU/ml with respect to the World Health Organization international standard 20-136. After vaccination, antibody responses were more rapid and of higher magnitude in those individuals who were seropositive at baseline. Natural infection with SARS-CoV-2 prior to enhanced PPE was significantly higher in DCPs than the regional population. Natural infection leads to a serological response that remains detectable in over 70% of individuals 6 mo after initial sampling and 9 mo from the peak of the first wave of the pandemic. This response is associated with protection from future infection. Even if serological responses wane, a single dose of the Pfizer-BioNTech 162b vaccine is associated with an antibody response indicative of immunological memory.

## Introduction

Seroepidemiological studies of health care workers define occupational risk of exposure to the severe acute respiratory syndrome coronavirus 2 (SARS-CoV-2) virus (Eyre et al. 2020; Houlihan et al. 2020; Shields, Faustini, Perez-Toledo, Jossi, Aldera, et al. 2020), and seropositivity is associated with protection from subsequent infection in high-exposure cohorts (Hanrath et al. 2020; Lumley et al. 2020). Such studies have guided public health planning, the design of health care services, and associated infection prevention protocols to mitigate risk and maintain essential care services during the pandemic. However, an absolute level of antibodies that confers protection for a given period of time remains unknown.

Dental care professionals (DCPs) represent a group of health care professionals thought to be at high risk of exposure to SARS-CoV-2 because they routinely operate within patients’ aerodigestive tract and undertake aerosol-generating procedures (AGPs). The risks of occupational exposure to and infection with SARS-CoV-2 in dental teams remains unclear despite studies evaluating aerosol generation and viral recovery in relevant bodily fluids (Chau et al. 2020; Allison et al. 2021). However, SARS-CoV-2 viral loads are estimated at 10^4^ to 10^8^ in saliva (Zhu et al. 2020), and high salivary viral loads are associated with poor outcomes. In situ hybridization, alongside 3-dimensional (3D) imaging, has also demonstrated active viral replication within salivary epithelial cells (Huang et al. 2021), implying a higher risk for DCPs working within the oral cavity. Many dental practices across the world closed in March 2020 and, in the United Kingdom, did not reopen until June/July 2020, when level 3 personal protective equipment (PPE) was in sufficient supply to enable resumption of AGPs for those in need of urgent dental care. The impact of this policy on patients remains unclear, as does the impact of enhanced PPE and infection control practices following resumption of routine dental care.

In June 2020, we recruited a cohort of 1,507 community- and hospital-based DCPs from the West Midlands region of the United Kingdom, following the first wave of the COVID-19 pandemic. Longitudinal follow-up of this cohort through the second wave of the COVID-19 pandemic and following the start of widespread vaccination of health care workers afforded a unique opportunity to study occupational risk factors in DCPs. It also provided insights into the rates of new infections after resumption of routine dental services under enhanced PPE protocols and enabled analysis of the durability of serological responses. Moreover, we were able to compare the early kinetics of serological responses following a single dose of the Pfizer-BioNTech 162b2 vaccine based on prior exposure to the virus. Furthermore, using World Health Organization (WHO) and National Institute for Biological Standards and Control (NIBSC) international reference material, we were able to define an anti–SARS-CoV-2 spike glycoprotein antibody concentration, arising following natural infection that was associated with protection from reinfection for 6 mo.

## Methods

### Study Recruitment

**Figure 1.**
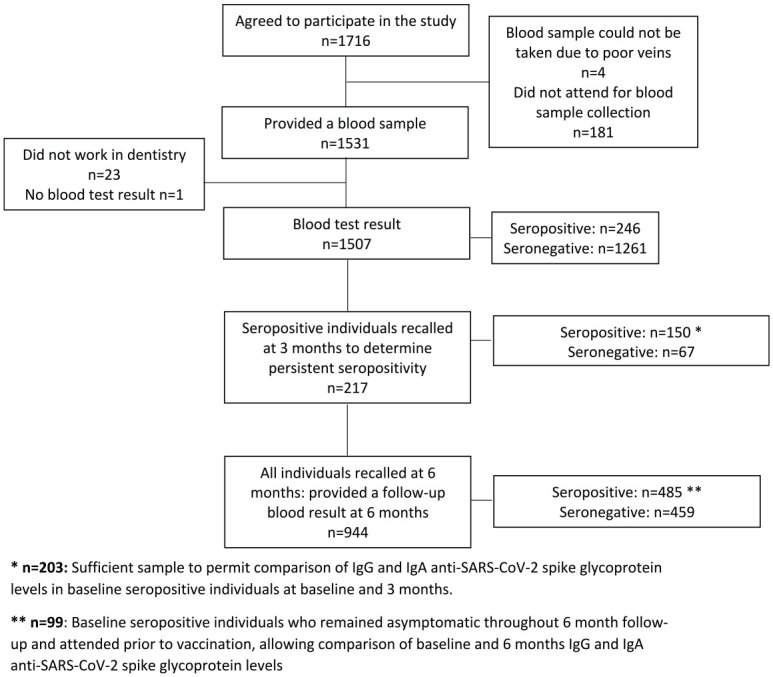
Participant flowchart through the study alongside headline serological data. SARS-CoV-2, severe acute respiratory syndrome coronavirus 2.

Registered general dental practitioners (GDPs) in the West Midlands area were invited by email to participate in a research study on SARS-CoV-2 antibody status in May 2020. GDPs were encouraged to disseminate this invitation to their wider dental teams. A total of 1,716 individuals registered their interest, of whom 1,535 attended the first study visit and provided informed written consent. Twenty-three individuals were excluded when they were found to not work in dentistry. In total, 1,507 participants volunteered a venous blood sample that was suitable for serological analysis at their first study appointment. Study participants also provided occupational and ethnodemographic data. The index of multiple deprivation (the official UK government measure of relative deprivation in England) in participants’ home postcode was sourced from 2019 UK Ministry of Housing, Communities and Local Government statistics (Ministry of Housing, Communities and Local Government 2019). Study data were collected and managed using REDCap electronic data capture tools hosted at the University of Birmingham (Harris et al. 2009). Individuals who were seropositive at baseline were recalled 3 mo later (September 2020) to study the persistence of their antibody response. All participants were recalled 6 mo after providing their baseline sample (January 2021). In total, 944 participants volunteered a repeat blood sample suitable for serological analysis (Fig. 1).

### Serological Analysis

Serological analysis was performed using a commercially available, CE-marked, IgGAM enzyme-linked immunosorbent assay (ELISA) that is optimized for seroprevalence studies and measures the total antibody response against the spike glycoprotein (product code: MK654; The Binding Site [TBS]). Briefly, this assay simultaneously measures any IgG, IgA, or IgM directed against the spike glycoprotein, facilitating detection of any antibody response against the antigen. Development of this assay was undertaken by the authors (AMS, SEF, AMC, MTD, AGR) at the University of Birmingham in collaboration with The Binding Site. Detailed descriptions of this assay, including its construction, validation, and verification, have been published previously (Cook et al. 2021; Faustini et al. 2021). The assay demonstrates 98.3% (95% confidence interval [CI], 96.4%–99.4%) specificity and 98.6% sensitivity (95% CI, 92.6%–100%) in detecting serological responses to the SARS-CoV-2 spike glycoprotein following polymerase chain reaction (PCR)–positive, nonhospitalized, mild-to-moderate coronavirus disease 2019 (COVID-19). Internal quality control material demonstrates an interassay coefficient of variance of 7.2% at the cutoff. Samples are run at a standard dilution of 1/40.

To provide greater detail on the composition of the total serological response and insight into the correlates of protective humoral immunity (rather than seroprevalence), the IgG and IgA responses against the spike glycoprotein were measured individually. To do this, the IgGAM ELISA protocol was modified. The antigen coating layer, serum dilution, and washing steps were unchanged from the original IgGAM protocol (above), but the detection layer employed polyclonal sheep-anti-human horseradish peroxidase (HRP)–conjugated antibodies directed against IgG (1:16,000) or IgA (1:2,000) individually, rather than in combination. For these assays, a cutoff ratio of 1.0 relative to the existing TBS cutoff calibrators was determined by plotting the pre-2019 negatives (*n* = 90) in a frequency histogram chart. Once the ratio cutoff was determined from the pre-2019 negatives, a cutoff multiplier of 1.0 and 0.71 was established for IgG and IgA, respectively. Further comparison of the properties and comparative performance of these assays relative to the IgGAM assay and others has also been published (Shields, Faustini, Perez-Toledo, Jossi, Allen, et al. 2020; Mohanraj et al. 2021).

### NIBSC and WHO Standards

In late 2020, the NIBSC developed international reference material (IRM) for the purposes of traceability and calibration of SARS-CoV-2 serological tests. These include NIBSC 20/136, the first WHO International Standard for anti–SARS-CoV-2 immunoglobulin (Mattiuzzo et al. 2020), and NIBSC 20/162. Serial dilutions of these IRMs were run in triplicate on the SARS-CoV-2 IgG assay described above. A receiver operator characteristics curve was constructed using baseline anti–SARS-CoV-2 IgG antibody levels and binary seropositivity/seronegativity at 6 mo as the outcome variable. In reference to the NIBSC standard, the minimum level of anti–SARS-CoV-2 IgG antibodies in baseline samples associated with protection for 6 mo was inferred, based on the original dilution of samples.

### Statistical Analysis

Data were analyzed in Stata 16 (StataCorp LLC) and Graph Pad Prism 9.0 (GraphPad Prism Software). With respect to demographic data, categorical characteristics were compared using a χ^2^ test and continuous characteristics compared using the Wilcoxon rank-sum test. The distribution of IgG ratios at different time points was compared using the Kolmogorov–Smirnov test with a false discovery rate approach set at 1% (Benjamini, Krieger, and Yekutieli method).

### Ethical Approval

The study was approved by the London-Camden and Kings Cross Research Ethics Committee (reference 20/HRA/1817). All participants provided written informed consent prior to enrollment in the study.

## Results

Following the first wave of the COVID-19 pandemic, the baseline seroprevalence of anti–SARS-CoV-2 spike glycoprotein antibodies in this cohort of DCPs was 16.3% (*n* = 246/1,507) (Table). Consistent with large community studies (Lavezzo et al. 2020), 60.2% of seropositive study participants (*n* = 148/246) reported symptomatic illness; 25.6% (*n* = 63/246) reported cough, 23.3% (*n* = 58/246) reported fever, and 39.0% (*n* = 96/246) reported a loss of sense of taste or smell. Ethnicity was a significant risk factor for seropositivity at baseline, with higher seroprevalence observed in individuals of Black ethnicity (35.0%), compared to those of Asian (18.8%) and White ethnicity (14.3%) (*P* = 0.018). Although based on a small sample size, these data are concordant with similar studies involving cohorts of non–dental health care professions (Eyre et al. 2020; Shields, Faustini, Perez-Toledo, Jossi, Aldera, et al. 2020) and with UK national data (Public Health England 2020a).

**Table.** Demographics of the Study Population.

Characteristic	All Participants	Seropositive	Seronegative	Seroprevalence, %	*P* Value
*n*	1507 (100)	246 (16.3)	1261 (83.7)	16.3	
Age, median (IQR), y	37 (29, 47)	36 (28, 46)	37 (29, 48)		0.130
Sex					
Male	371 (24.6)	56 (22.8)	315 (25.0)	15.1	0.461
Female	1136 (75.4)	190 (77.2)	946 (75.0)	16.7	
Ethnicity					
White	830 (55.1)	119 (48.4)	711 (56.4)	14.3	0.020
Asian	584 (38.8)	110 (44.7)	474 (37.6)	18.8	
Black	20 (1.3)	7 (2.9)	13 (1.0)	35.0	
Mixed	39 (2.6)	7 (2.9)	32 (2.5)	18.0	
Other	34 (2.3)	3 (1.2)	31 (2.5)	8.8	
Index of multiple deprivation rank, median (IQR)	11,750 (3,717, 21,688)	8,238 (3,240, 14,408)	12,081 (3,858, 21,795)		0.004
Diabetic					
Yes	21 (1.4)	4 (1.6)	17 (1.4)	19.1	0.734
No	1,486 (98.6)	242 (98.4)	1,244 (98.6)	16.3	
Other medical conditions					
Yes	354 (23.3)	55 (22.4)	299 (23.7)	15.5	0.647
No	1,153 (76.7)	191 (77.6)	962 (76.3)	16.6	
Smoking					
Never	1,100 (73.0)	193 (78.5)	907 (71.9)	17.6	0.007
Former	250 (16.6)	41 (16.7)	206 (16.6)	16.4	
Current	157 (10.4)	12 (4.9)	145 (11.5)	7.6	
Occupation					
Dentist	687 (45.6)	115 (46.8)	572 (45.4)	16.7	0.398
Dental nurse	528 (35.0)	89 (36.2)	439 (34.8)	16.9	
Dental hygienist/therapist	116 (7.7)	18 (7.3)	98 (7.7)	15.5	
Receptionist	80 (5.3)	5 (2.0)	75 (6.0)	6.3	
Clinical dental technician	2 (0.1)	0 (0.0)	2 (0.2)	0.0	
Practice manager	51 (3.4)	11 (4.5)	40 (3.2)	21.6	
Other dental health care occupation	22 (1.5)	5 (2.0)	17 (1.4)	22.7	
Auxiliary staff in dental practice/hospital/clinic	13 (0.9)	2 (0.8)	11 (0.9)	15.4	
Other (no detail provided)	8 (0.5)	1 (0.4)	7 (0.6)	12.5	

Values are presented as *n* (%) for categorical and binary characteristics and compared using a χ^2^ test. Medians (interquartile ranges [IQRs]) are presented for continuous characteristics and compared using the Wilcoxon rank sum test.

There were no differences in seroprevalence between different DCPs; receptionists, who do not have direct patient contact, had the lowest baseline seroprevalence (6.3%), a finding concordant with estimates of seroprevalence in the general population of the West Midlands at the time of baseline sampling (Public Health England 2020b). Current smoking was associated with a lower seroprevalence compared to never-smokers or ex-smokers (7.6% vs. 16.4% vs. 17.6%, *P* = 0.007). Deprivation was associated with a higher seroprevalence: the median index of multiple deprivation rank was 8,238 (interquartile range [IQR], 3240, 14,408) for seropositive individuals compared to 12,081 (IQR, 3,858, 21,795) for those that were seronegative (*P* = 0.004).

**Figure 2.**
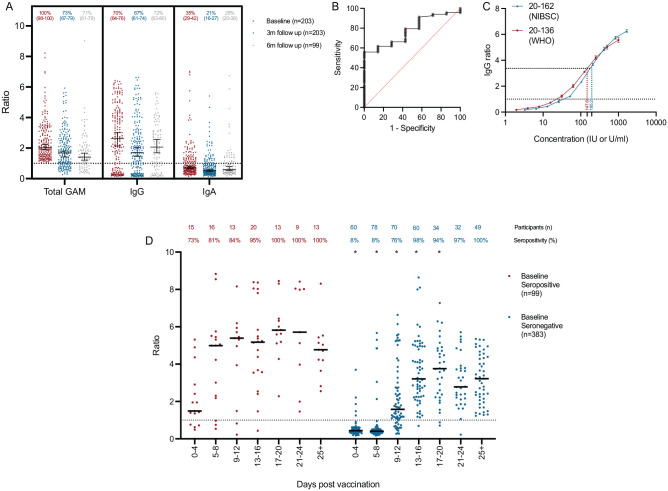
Longitudinal seroprevalence and vaccine responses in dental care professionals. (A) Total anti–severe acute respiratory syndrome coronavirus 2 (SARS-CoV-2) spike glycoprotein antibodies (Total GAM) and IgG and IgA anti–SARS-CoV-2 spike glycoprotein antibodies measured at baseline and 3-mo and 6-mo follow up. At 6 mo, only individuals who had not been vaccinated are shown. Data are provided as a ratio of the level of antibody compared to the cutoff calibrator set at 1.0. Percentages of individuals above the assay cutoff at each time point are also provided above each column with 95% confidence intervals provided below in parentheses. Median and 95% confidence intervals are also shown. (**B**) Receiver operator characteristic curve describing the relationship between baseline anti–SARS-CoV-2 spike glycoprotein IgG ratio and binary IgG seropositivity/seronegativity in unvaccinated, non-reinfected individuals at 6-mo follow up. Area under the curve = 0.77, *P* = 0.01 (*n* = 75). (**C**) International reference materials NIBSC 20-136 (World Health Organization) and 20-162 were run in triplicate serial dilutions and the IgG ratio determined. The minimum IgG ratio associated with guaranteed ongoing seropositivity 6 mo from baseline is shown by the red and blue dotted lines. (**D**) Kinetics of total antibody response in 490 individuals following a single dose of the Pfizer-BioNTech vaccine. *Demonstrates a significant difference (*P* < 0.05) between the distributions of IgGAM ratios at each time point following vaccination between individuals who were seropositive and seronegative at baseline as determined by Kolmogorov–Smirnov test. Percentage of individuals above the assay cutoff at each time point is also provided.

The cohort was followed longitudinally: individuals who were seropositive at baseline were rebled at 3 mo to study the durability of serological responses (Fig. 2A). Seventy percent of individuals continued to have a detectable serological response against the spike glycoprotein at 3 mo; in a subgroup of 99 individuals who were seropositive at baseline, remained asymptomatic throughout follow-up, and reattended at 6 mo prior to vaccination, the original serological response remained detectable in 71%. Individual IgG and IgA responses were also measured in those who were seropositive to document the composition of the total serological response. Antispike glycoprotein IgG and IgA responses were detectable in 73% and 35% of individuals at baseline, reducing to 67% and 21% at 3 mo and 72% and 28% at 6 mo, respectively. The discordance between seropositivity defined by the detection of the total antibody response (IgGAM) against the SARS-CoV-2 spike glycoprotein, compared to the IgG isotype, arises from the optimization of the IgGAM assay for sensitivity in seroepidemiological studies (see Methods).

In total, 74.1% (*n* = 1,116/1,507) of the cohort returned questionnaires regarding SARS-CoV-2 infections at 6 mo, and 62.6% (*n* = 944/1,507) were rebled. In this cohort, 94 PCR-positive SARS-CoV-2 infections were reported by study participants, representing an overall infection risk of 8.4%. The risk of infection was 9.7% in participants who were seronegative at baseline, compared to 2.9% in individuals who were seropositive (*P* = 0.001). As seropositivity at baseline in June 2020 could only be accounted for by SARS-CoV-2 infection, these data suggest that the emergence of antibodies following natural infection is associated with a 75% risk reduction for future infection (risk ratio, 0.25; 95% CI, 0.09–0.73, adjusted for age, sex, ethnicity, and smoking).

To further investigate the phenomenon of reinfection in participants who were seropositive at baseline using the IgGAM assay, participants’ individual IgG and IgA responses against the SARS-CoV-2 spike glycoprotein were reviewed. Symptomatic reinfections only occurred in the absence of a specific, detectable antispike glycoprotein IgG response, either due to lack of an initial IgG response (*n* = 6) or loss of that response over time (*n* = 1). Thus, to determine an absolute level of IgG anti–SARS-CoV-2 antibodies associated with ongoing seropositivity at 6 mo, we reviewed the baseline antispike glycoprotein IgG levels of the 75 participants who were IgG positive at baseline that had been resampled prior to vaccination at 6 mo (Fig. 2B). An IgG ratio greater than 2.95 conferred a likelihood ratio of 2.32 of IgG seropositivity at 6 mo (sensitivity, 64.7% [95% CI, 54.3%–76.3%]; specificity, 71.4% [95% CI, 35.9%–94.2%]); no participant with a baseline IgG ratio greater than 3.36 was IgG seronegative at 6 mo (sensitivity, 55.8% [95% CI, 44.1%–67.1%]; specificity, 100% [95% CI, 64.5%–100.0%]). In reference to the first WHO standard for SARS-CoV-2 immunoglobulin (NIBSC 20/136) and the original dilution of the baseline samples, we estimate that the minimum level of anti–SARS-CoV-2 spike glycoprotein IgG antibodies necessary to confer 6 mo of protection from infection is 147.6 IU/mL (Fig. 2C). Studies using the NIBSC standard 20/162 generated a similar estimate of 195.2 U/mL.

In total, 944 participants were rebled in January 2021 following 6 mo of follow-up. Through natural infection and vaccination, the overall seroprevalence had risen to 51.4% (*n* = 485/944). A total of 329 participants who were seronegative at baseline provided a sample prior to vaccination. Seroconversion had occurred in 19.7% (*n* = 65/329) of these participants, which could only be attributable to natural infection; 38.4% (*n* = 25/65) of these seroconversion events occurred asymptomatically, and the remainder occurred in association with an illness consistent with COVID-19, 87.5% of which were proven by PCR.

In total, 53.9% (*n* = 509/944) of participants had already received at least 1 dose of a SARS-CoV-2 vaccine (Oxford/AstraZeneca, *n* = 20; Pfizer-BioNTech 162b2, *n* = 484; unknown, *n* = 5). The serological responses of individuals receiving a single dose of the Pfizer-BioNTech 162b2 SARS-CoV-2 were analyzed based on prior exposure to the virus defined by either positive baseline serology or PCR-proven infection during the follow up period (Fig. 2D). Vaccination on the background of prior exposure to the virus was associated with a more rapid and quantitatively greater total antibody response against the SARS-CoV-2 spike glycoprotein, consistent with the boosting of immunological memory. In immunologically naive participants, 97.7% seroprevalence was achieved among vaccine recipients sampled at least 12 d after immunization.

## Discussion

Consistent with other studies, we demonstrate that natural infection with SARS-CoV-2 is generally associated with robust and durable serological responses (Wajnberg et al. 2020; Dan et al. 2021). Furthermore, in this community-based cohort of DCPs, we corroborate the hospital-based studies of Lumley et al. (2020) and Hanrath et al. (2020) in demonstrating that seropositivity arising from natural infection is associated with longitudinal protection from reinfection with SARS-CoV-2. In our study, we observed that symptomatic reinfections only occurred in individuals who lacked a robust IgG response and thus, by relating initial antispike glycoprotein IgG levels to the WHO first international reference material for anti–SARS-CoV-2 immunoglobulin (NIBSC 20/136), define a putative antibody level of 147.6 IU/mL that affords a minimum of 6 mo of protection from reinfection. Critically, only 5.3% of the cohort developed an IgG response that exceeded this threshold following the first wave of the UK pandemic. This suggests that natural infection alone is unlikely to generate meaningful, durable herd immunity.

Clinically, real-world data that relate protection from infection with antibody binding in an in vitro assay are invaluable. Further longitudinal studies in cohorts of individuals following natural infection and vaccination will be necessary to replicate these findings using assays that employ alternative target SARS-CoV-2 antigens, such as the receptor binding domain, or nucleocapsid. Essential to this process will be a comprehensive understanding of the performance of assays used to determine SARS-CoV-2 seropositivity and quantify responses. This study highlights the potential challenges in this process. Antispike glycoprotein IgG responses were detectable in 70% of seropositive individuals at baseline, and consistent with other studies, IgG seropositivity remains stable over time. However, the combined IgGAM assay, optimized to determine seroprevalence, rather than durable immunity (Cook et al. 2021), detects a further 30% of individuals who mount only modest, transient, serological responses suggestive of exposure to the virus but not associated with durable humoral immunity.

Vaccination is the most cost-effective and efficacious public health intervention of modern times. In the United Kingdom, the rapid deployment of the Pfizer-BioNTech 162b2 SARS-CoV-2 vaccination coincided with a planned 6-mo follow-up of this cohort, affording a unique opportunity to investigate the early serological response to vaccination. Following a single dose of vaccine in immunologically naive recipients, SARS-CoV-2 antibodies were detectable in over 95% of individuals 12 d after vaccination and persisted beyond 25 d post vaccination. In keeping with other contemporaneous studies, we also demonstrate that prior viral infection leads to a more rapid and robust antibody response, consistent with secondary immunological responses (Krammer et al. 2021; Stamatatos et al. 2021). The nature and duration of immunity in these cohorts will be critical to understand as the COVID-19 pandemic progresses, particularly with respect to the efficacy of vaccination strategies (single dose, multiple doses, vaccine combinations) and in relation to novel viral variants of concern.

Finally, in this community-based cohort of over 1,500 individuals, we demonstrate that DCPs had a significant occupational risk of exposure to the SARS-CoV-2 virus prior to the closure of routine dentistry, which occurred on March 25, 2020 in England. The overall baseline seroprevalence in this study of 16.3% exceeded that of the general population in the West Midlands region (6%–7%) in June 2020 (Public Health England 2020a). Between August and October 2020, during the early stages of the second wave of the UK pandemic, the percentage of patients attending general dental practices who were asymptomatically or presymptomatically infected with SARS-CoV-2 peaked at 1.7% (Conway et al. 2021), providing evidence that dental care professionals are directly exposed infected individuals during the course of their work. The observation that the seroprevalence among dental practice receptionists (6.3%), who have no direct patient contact, was comparable to the general population supports the hypothesis that the increased occupational risk we have observed arose from close, clinical exposure to patients. Although dental practice managers were found to have a higher seroprevalence (21.6%), this may be accounted for by the fact that, in the United Kingdom, practice managers are typically senior dental nurses who remain clinically active as part of their role. To further contextualize the risk faced by DCPs, the overall seroprevalence of a mixed cohort of health care workers employed by University Hospitals Birmingham NHS Foundation Trust, the largest tertiary care provider in the West Midlands, was 24.4% in late April 2020, suggesting overall risk of viral exposure in general dental practice was lower than in those exposed to acutely unwell COVID-19 patients (Shields, Faustini, Perez-Toledo, Jossi, Aldera, et al. 2020).

Concordant with other studies in health care workers and national statistics, non-White ethnicity and residence within more socially deprived neighborhoods were both associated with greater seroprevalence (Eyre et al. 2020; Shields, Faustini, Perez-Toledo, Jossi, Aldera, et al. 2020; Public Health England 2020b). Our observation that current smokers had significantly lower SARS-CoV-2 seroprevalence was unexpected. The effect of smoking status on the risk of symptomatic infection remains unclear, with studies in health care workers and the general population producing different conclusions (Hopkinson et al. 2021; Kua et al. 2021). In the United Kingdom, smoking is forbidden within indoor public spaces; thus, current smokers may have reduced exposure to the virus by virtue of being outside. Equally, hematogenous dissemination of the virus from oral salivary reservoirs to the pulmonary vasculature has been proposed as a potential mechanism to explain the severe lung disease observed in COVID-19 (Lloyd-Jones et al. 2021). Nicotine is a potent vasoconstrictor and immunomodulator; it may directly dampen immune responses, reducing the magnitude of serological responses and hampering their subsequent detection, or indirectly reduce systemic immune responses by preventing hematogenous spread, and further research is necessary to understand the observation we have made. Nonetheless, multiple studies demonstrate that individuals with preexisting pulmonary disease have poorer outcomes from COVID-19 (Docherty et al. 2020; Williamson et al. 2020), and the overriding public health message must be that of the benefits of smoking cessation.

Dentistry reopened in England on June 8, 2020, under new Public Health England guidance for infection control, including FFP3 masks, eye protection, and gowns for AGPs, although most practices did not reopen until July 2020. Seroprevalence across the West Midlands region increased by 12.3% between June 2020 and January 2021 (Office of National Statistics 2021); the risk of PCR-proven infection in seronegative DCPs in our study during this time was 11.7%. This implies that the enhanced PPE and infection control practices appeared effective in reducing risk of occupational exposure of DCPs to SARS-CoV-2 to background population levels. Further studies are necessary to comprehensively understand whether these comparative statistics represent a true lowering of exposure rates of DCPs following reopening of general dental practices and the additional precautions taken to ensure practices became COVID-19 secure.

## Author Contributions

A.M. Shields, T. Dietrich, I.L.C. Chapple, contributed to conception, design, and data analysis, drafted and critically revised the manuscript; S.E. Faustini, C.A. Kristunas, A.M. Cook, C. Backhouse, L. Dunbar, D. Ebanks, B. Emmanuel, M.T. Drayson, contributed to data analysis, critically revised the manuscript; E. Crouch, A. Kröger, J. Hirschfeld, P. Sharma, R. Jaffery, S. Nowak, S. Gee, contributed to conception and design, critically revised the manuscript; A.G. Richter, contributed to conception, design, and data analysis, critically revised the manuscript. All authors gave final approval and agree to be accountable for all aspects of the work.
